# Stabilization of the genome of the mismatch repair deficient *Mycobacterium tuberculosis *by context-dependent codon choice

**DOI:** 10.1186/1471-2164-9-249

**Published:** 2008-05-28

**Authors:** Roger M Wanner, Carolin Güthlein, Burkhard Springer, Erik C Böttger, Martin Ackermann

**Affiliations:** 1 Institut für Medizinische Mikrobiologie, Universität Zürich, Gloriastrasse 30/32, CH-8006 Zürich, Switzerland; 2 Institute of Medical Microbiology and Hygiene Graz, Austrian Agency for Health and Food Safety, Beethovenstrasse 6, A-8010 Graz, Austria; 3 Institute of Integrative Biology, ETH Zurich, Universitaetsstrasse 16, CH-8092 Zürich, Switzerland

## Abstract

**Background:**

The rate at which a stretch of DNA mutates is determined by the cellular systems for DNA replication and repair, and by the nucleotide sequence of the stretch itself. One sequence feature with a particularly strong influence on the mutation rate are nucleotide repeats. Some microbial pathogens use nucleotide repeats in their genome to stochastically vary phenotypic traits and thereby evade host defense. However, such unstable sequences also come at a cost, as mutations are often deleterious. Here, we analyzed how these opposing forces shaped genome stability in the human pathogen *Mycobacterium tuberculosis*. *M. tuberculosis *lacks a mismatch repair system, and this renders nucleotide repeats particularly unstable.

**Results:**

We found that proteins of *M. tuberculosis *are encoded by using codons in a context-dependent manner that prevents the emergence of nucleotide repeats. This context-dependent codon choice leads to a strong decrease in the estimated frame-shift mutation rate and thus to an increase in genome stability.

**Conclusion:**

These results indicate that a context-specific codon choice can partially compensate for the lack of a mismatch repair system, and helps to maintain genome integrity in this pathogen.

## Background

*M. tuberculosis *is the causative agent of tuberculosis. It is one of the most harmful pathogens causing 10 million new infections and 1.6 million deaths every year [1]. There is a considerable interest in understanding the genome evolution of this pathogen, as this can further our understanding of pathogenicity and contribute to the development of new vaccines [2-5]. Here we focus on one important aspect of genome evolution: structural properties of the DNA sequence that determine the local mutation rate.

The rate at which a stretch of DNA mutates is influenced by the nucleotide sequence itself. Certain sequences are inherently prone to errors during replication and gene expression, while other sequences are more stable. Particularly unstable are simple sequence repeats, which consist of short motifs of up to six nucleotides that are repeated several times [6]. Simple sequence repeats are prone to length variation during replication because of DNA-polymerase slippage [7]. The mutation rate in simple sequence repeats is much higher than in non-repeated sequences [8,9], and increases with the number of repeats [7,10-13]. If simple sequence repeats are located in coding regions, and if they consist of motifs whose length is not a multiple of three, then a change in the number of repeats leads to a frame-shift mutation, and thereby to a complete loss of the amino acid sequence information.

Some organisms, and particularly microbial pathogens, contain conspicuously long simple sequence repeats in coding regions [14]. For example, the bacteria *Haemophilus influenzae *[15], *Neisseria meningitidis *[16], *and Campylobacter jejuni *[17] contain mononucleotide, dinucleotide and tetranucleotide repeats in genes that are involved in interactions with the host. The most plausible biological function of these repeats is that they promote phenotypic variation among otherwise clonal cells by altering protein expression profiles at a high rate [18]. This phenotypic variation might allow pathogens to evade the immune system, as well as to increase the probability that a fraction of a clonal population survives changing conditions [19].

However, unstable nucleotide sequences also come at a cost, as many mutational changes are detrimental. Recent studies suggested that simple sequence repeats are avoided in genomes of non-pathogenic organisms [20-22]. It is thus currently not clear whether unstable nucleotide sequences are ubiquitous among microbial pathogens, or whether selection for stability usually also prevails in these genomes and leads to a bias against simple sequence repeats. Given the significance of *M. tuberculosis *as pathogen, it is important to investigate whether the genome of this microorganism is biased towards stability or instability.

Mycobacteria are especially prone to polymerase slippage in simple sequence repeats, for two reasons. First, their high G+C content (65.6% in *M. tuberculosis*; [2]) makes the emergence of repeats more likely. Second, they lack a mismatch repair system (MMR) [23] or other orthologous repair systems that could compensate for this deficiency [2,24,25]. MMR deficiency leads to a high mutation rate in simple sequence repeats, and especially in mononucleotide repeats [26], and it has been shown experimentally that in mycobacteria, the frame-shift rate in mononucleotide repeats is particularly high [23].

Here, we analyzed whether proteins in *M. tuberculosis *are encoded in a manner that prevents or promotes the emergence of unstable sequences. The genome of *M. tuberculosis *contains a number of nucleotide repeats, and some of these repeats are polymorphic between related genomes [27], raising the question whether nucleotide repeats in this organism are involved in genomic variation. However, a global analyis suggested that long repeats are generally less frequent than expected if nucleotides were randomly distributed [28]. We investigated here the causes and consequences of this under-representation. Focusing on short nucleotide repeats, which are a particularly important determinant of stability, we found that a context-dependent codon choice in the genome of *M. tuberculosis *leads to a bias against these unstable sequences, and that this leads to a strong increase in estimates of genetic stability.

## Results and discussion

### Comparing frequencies of mononucleotide repeats in coding regions and intergenic regions

A first insight into the distribution of short nucleotide repeats can be gained from the comparison of coding and intergenic regions. The frequency of repeats in a particular region of the genome is determined by the combination of mutational processes and selection. Mutational processes are expected to be similar in coding and intergenic regions (except for transcription-mediated mutations and repair [29]). In contrast, selection is generally assumed to be stronger on coding regions than on intergenic regions. If selection leads to a bias for or against repeats, one would expect this bias to be stronger in coding regions than in intergenic regions. We tested this by comparing observed and expected occurrences of repeats in these two regions. We focused on mononucleotide repeats, because they are particularly unstable and more frequent than other types of repeats.

**Figure 1:**
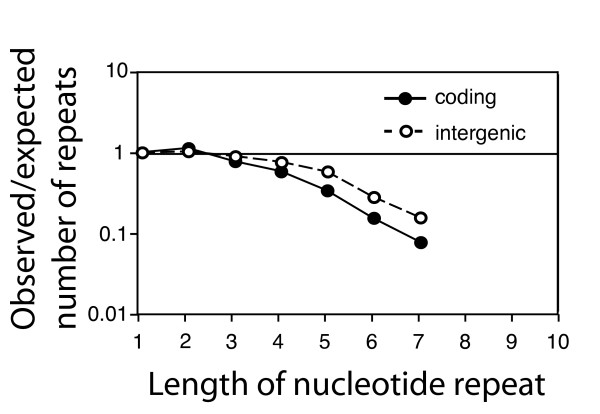
**Mononucleotide repeats are less frequent in coding regions than in intergenic regions in the genome of *M. tuberculosis*.** The figure shows the ratio between total number of observed and expected mononucleotide repeats in coding (filled circles and solid line) and intergenic regions (open circles and dashed line). The expected values have been calculated under the null model that nucleotides are randomly distributed in each of these two compartments, and occur at the frequencies observed in each compartment. For repeats of length three to six nucleotides, the under-representation of mononucleotide repeats is significantly stronger in coding regions than in intergenic regions (at p < 0.0001, chi square test).

We first determined the number of mononucleotide repeats that would be expected if nucleotides were randomly distributed within each region, and then compared this number to the actually observed frequencies of repeats. We found that repeats of three nucleotides and more are less frequent than expected in both coding and intergenic regions (Figure 1). The bias increases with increasing repeat length, and is stronger in coding than in intergenic regions (at p < 0.0001 for repeats of length 3 to 7; chi-square test). The latter finding is consistent with the hypothesis that the bias against repeats might be a consequence of selection against unstable sequences. We note that this result has to be interpreted cautiously, because regulatory and structural functions in intergenic regions [30,31] impose constrains that can bias the frequency of repeats.

### Context-dependent codon choice in *M. tuberculosis *limits the emergence of nucleotide repeats

In this first comparison, we calculated the expected frequencies of mononucleotide repeats under the assumption that nucleotides are randomly distributed. This null-model is appropriate for comparisons involving intergenic regions. In coding regions, however, a null-model that preserves the amino acid sequence and gene-specific codon frequencies is more adequate [22]. Such a modified null-model accounts for constraints on the DNA sequence imposed by the need to encode a particular protein, and it accounts for regional variation in GC-content and codon usage. With such a null-model, one can ask: if proteins would be encoded by using codons independently of the sequence context, how many repeats would be expected? If the actually observed frequencies are higher than these expected frequencies, it can be concluded that codons are used in a context-dependent manner that promotes the emergence of repeats. If, in contrast, observed frequencies are lower than expected frequencies, one can conclude that in the actual genome, codons are used in a context-dependent manner that prevents the emergence of repeats.

We thus re-analyzed coding regions by using this modified null-model. For each gene, we determined the codon frequencies, and then re-arranged the codons while preserving the amino acid sequence. Such a re-arrangement is possible because the genetic code is degenerate; as most amino acids are encoded for by more than one codon, a given amino acid sequence can be encoded for by different nucleotide sequences. For each gene, we generated 100 randomized sequences. Then we asked whether the observed nucleotide sequence differs from the random sequences in the frequency of short nucleotide repeats, and thus in terms of stability

**Figure 2:**
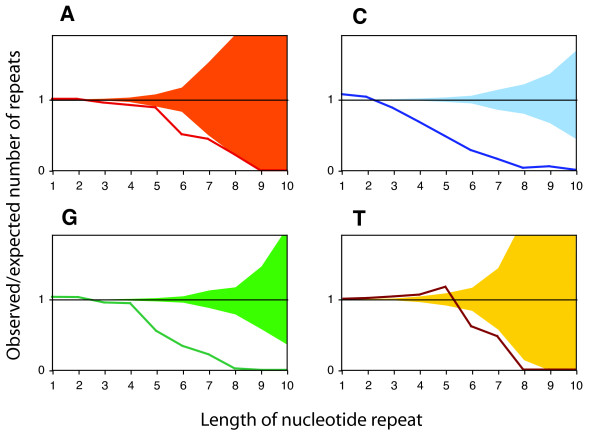
**Proteins of *M. tuberculosis *are encoded in a way that minimizes the emergence of mononucleotide repeats.** The lines depict the ratio between the observed and expected number of mononucleotide repeats (summed over all genes in the genome) as a function of their length. The expected numbers were calculated with a null-model that conserved the amino acid sequence and the gene-specific codon frequencies. The areas comprise 95% of the data from the randomized genomes. For repeats of three (for A, C and G) or six (for T) nucleotides and longer, the lines lie below this area, indicating that such repeats are significantly under-represented.

We again first focused on mononucleotide repeats, and found that these repeats are strongly under-represented in coding regions of *M. tuberculosis *(Figure 2 and Additional File 1). The under-representation increases with increasing repeat length. Furthermore, the bias is stronger against runs of cytosine and guanine than against runs of adenine and thymine. This pattern can be explained by the fact that mononucleotide repeats of cytosine and guanine have a higher mutation rate than repeats of adenine and thymine [22,32,33].

**Figure 3:**
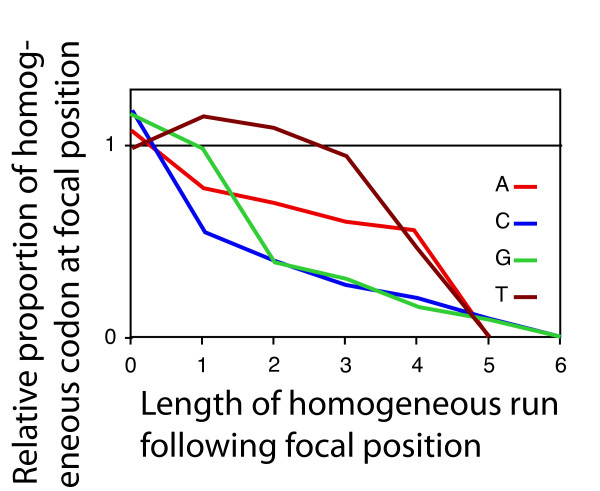
**The under-representation of long mononucleotide repeats in coding regions of *M. tuberculosis *is a consequence of a context-dependent codon choice.** Codons consisting of three identical nucleotides ('homogeneous codons') are avoided at positions followed by one or more nucleotides of the same type. The under-representation increases with increasing number of identical nucleotides following. Each line represents data for one type of nucleotide (indicated by the line color).

Importantly, the bias against mononucleotide repeats depicted in Figure 2 is not a consequence of an avoidance of certain codons; the randomized sequences have the same codon frequencies as the observed sequence. Rather, as discussed above, the bias results from a context-dependent codon choice. For example, the amino acid proline can be encoded by CCA, CCC, CCG and CCT. The codon CCC is used often (at 34%) at positions that are *not *followed by a cytosine. At positions followed by one cytosine, the frequency of CCC drops to 16%. With every additional cytosine following, the frequency decreased further (Figure 3). This decrease is statistically significant (p < 0.0001, logistic regression). This same pattern holds for the codons AAA and GGG (and is also significant at p < 0.0001; logistic regression). For TTT, the pattern is slightly different; the relative frequency of TTT first increases at positions followed by one thymidine, and then decreases at positions followed by more than one thymidine.

### Quantifying the stabilizing effect of context-dependent codon choice

As nucleotide repeats are prone to frame-shift mutations, the under-representation of mononucleotide repeats in the genome of *M. tuberculosis *is expected to lead to a decrease in the mutation rate. We sought to quantify the magnitude of this effect. First, we estimated the frame-shift mutation rate in mononucleotide repeats in coding regions of the *M. tuberculosis *genome. This estimate was based on published measurements of the frame-shift mutation rate in mononucleotide repeats in *Mycobacteria *and other bacteria (see Methods). Then, we estimated the frame-shift mutation rate that would be expected if codons were used at random, irrespective of the local context. To do so, we analyzed the randomized genomes, where codons were used in a context-independent manner, and estimated the frame-shift mutation in mononucleotide repeats in the same way as we did for the actual genome of *M. tuberculosis*. The ratio between estimated frame-shift mutation rates in the real and in the randomized genomes gave us an estimate for the magnitude of the stabilizing effect of context-dependent codon choice in the genome of *M. tuberculosis*.

**Figure 4:**
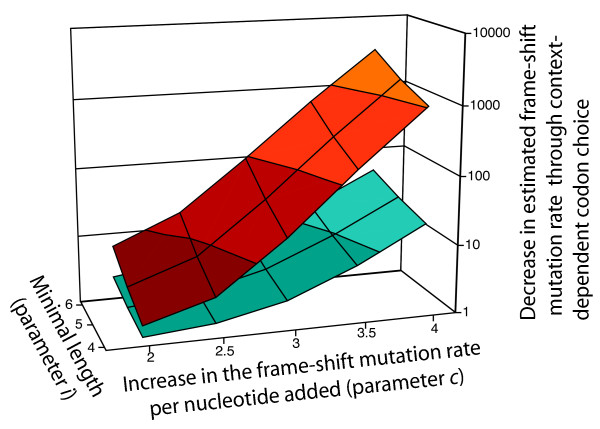
**Context-dependent codon choice leads to a strong decrease in the estimated frame-shift mutation rate in mononucleotide repeats in protein-coding genes in *M. tuberculosis *(upper area, red), and to a weaker decrease in *E. coli *(lower area, blue).** We estimated the frame-shift mutation rate in the real genomes, and in randomized genomes without context-dependent codon choice. The ratio between these two values is a measure for the degree by which context-dependent codon choice stabilizes the genome, and is displayed on the z-axis. Increasing lightness of the colors indicate increasing values on the z-axis. This ratio was calculated for different combinations of two biological parameters – the increase in the frame-shift mutation rate per nucleotide added to a mononucleotide repeat (parameter *c*, x-axis), and the minimal length for a mononucleotide repeat to exhibit a significant frame-shift mutation rate (parameter *i*, y-axis; see Methods). Published data suggest that in MMR-deficient bacteria *c *is close to 3 and *i *is about 4. For this combination, context-dependent codon choice leads to a decrease in the frame-shift mutation rate in mononucleotide repeats of a factor of about 20 in *M. tuberculosis *and of a factor of 3 in *E. coli*.

This ratio was sensitive to the mutation rate parameters retrieved from the literature, and we thus carried out the analysis for a range of parameter values. This analysis showed that the stabilizing effect of context-dependent codon choice was surprisingly strong: for a plausible range of biological parameters, the estimated frame-shift mutation rate in mononucleotide repeats in the randomized sequences was between 10 and 100 times higher than in the real sequence (Figure 4). For the parameter values that we considered most plausible (see Methods), the randomized genomes had an estimated frame-shift mutation rate that was about 20 times higher than the estimate for the real genome.

In fact, without context dependent codon choice, one would expect that frame-shift mutations in mononucleotide repeats would be the most frequent type of mutations in the *M. tuberculosis *genome. For the most plausible parameter values, we estimated that in the absence of context-dependent codon choice, the probability to acquire a frame-shift mutation in a mononucleotide repeat in a protein-coding gene would be 3% per replication (see Additional File 2). This value is about ten times higher than the *total *number of mutations expected per genome and replication in DNA-based microbes [34]. This means without bias against mononucleotide repeats, frame-shift mutations would happen very frequently, and they would constitute a very large fraction of all mutations that occur. Context dependent codon choice reduces the probability to acquire a frame-shift mutation in a mononucleotide repeat in a protein-coding gene by a factor of 20 to about 0.15% per replication (again with the parameter values that we regarded as most plausible, see Methods and Additional File 2). This indicates that context-dependent codon choice has a substantial impact on genome stability of *M. tuberculosis*.

We carried out the same analysis for the genome of the non-pathogenic *Escherichia coli *strain K12. Previous studies reported a bias against mononucleotide repeats for this organism, and attributed this bias to selection for stability [22]. Interestingly, the estimated stabilizing effect of context-dependent codon choice was much smaller for *E. coli *than for *M. tuberculosis *(Fig. 4). For the combination of parameters that we considered most plausible for *M. tuberculosis*, the estimated mutation rates for the real and the randomized genomes of *E. coli *differed by less than a factor of three (compared to a factor of 20 for *M. tuberculosis*). One possible explanation for this difference is that in the mismatch-deficient *M. tuberculosis*, selection against unstable sequences is stronger than in *E. coli*, and that this results in a stronger stabilization of the genome by context-dependent codon choice.

### Observed and expected frequencies of dinucleotide, trinucleotide and tetranucleotide repeats

In addition to mononucleotide repeats, tetranucleotide repeats, and to some extent also dinucleotide repeats, are other important determinants of the local mutation rate; they are also involved in phase variation in a number of pathogenic bacteria [35]. It is thus important to investigate whether context-dependent codon choice also leads to a bias against these two other types of repeats. To determine the expected frequencies of di- and tetranucleotide repeats, we again used a null model that conserved the amino acid sequence and the gene-specific codon frequencies. Then, we compared these expected frequencies of di- and tetranucleotide repeats to the actually observed frequencies.

Dinucleotide repeats are generally rare in the genome of *M. tuberculosis*, and the observed numbers are close to expected. There are twelve different types of dinucleotides (discounting AA, CC, GG and TT, which are also mononucleotide repeats). The longest observed dinucleotide repeats are of length five; there is a total of 41 occurrences of AC_5_, CA_5_, CG_5 _and GC_5_. By analyzing all dinucleotide repeats of length two and longer, we detected 27 cases where the observed number is significantly different from the expectation; in 19 of these 27 cases, the observed number is lower than the expected number. In the remaining eight cases, the expected number is lower than the observed number (see Additional File 1).

The results for tetranucleotide repeats are similar. There are 240 tetranucleotides that are not also mono- or dinucleotide repeats. The longest observed tetranucleotide repeats are of length four (two occurrences of GGCC_4_). There are 88 cases where the observed frequency of tetranucleotide repeats (of length two and longer) is significantly different from the expectation, and in 51 of those the observed number is lower than the expected number (see Additional File 1). These results indicate that context-dependent codon choice leads to some bias against di- and tetranucleotide repeats, but this bias is smaller than for mononucleotide repeats. This might be because mononucleotide repeats tend to have higher frame-shift mutation rates than other repeats, and tend to be more de-stabilized by the absence of a mismatch repair system [36].

We also analyzed observed and expected frequencies of trinucleotide repeats. These repeats differ in one important aspect from mono-, di- and tetranucleotide repeats: a gain or loss of one or more elements in a trinucleotide repeat does not lead to a frame-shift mutation; it only changes the length of the protein by one or a few amino acids, which is most likely much less deleterious than a frame-shift mutation. It was thus interesting to ask whether the bias against trinucleotide repeats is smaller or even absent. This is indeed the case; there are 83 cases where the observed frequency of trinucleotide repeats (of length two and longer) is significantly different from the expectation, and in only 37 of those the observed number is lower than the expected number (see Additional File 1); in other words, there is no evidence for a bias against trinucleotide repeats. As will be discussed in the next section, this is relevant for understanding the ultimate causes of the under-representation of mono-, di- and tetranucleotide repeats in the genome of *M. tuberculosis*.

### Selection or a neutral process?

The under-representation of mono-, di- and tetranucleotide repeats in the genome of *M. tuberculosis *could either result from selection for stable nucleotide sequences, or it could result from a neutral process [37]. A candidate for a neutral process is a deletion bias in nucleotide repeats. If deletions in short nucleotide repeats are more frequent than insertions, then repeats would decrease in size over the course of generations, and long repeats would become rare. However, in mono-, di- and tetranucleotide repeats, most deletions result in frame-shift mutation, which are usually deleterious; only deletions of a multiple of three units retain the reading frame. As most mutations in nucleotide repeats involve only one or two units [36], a deletion bias might not be a likely explanation for the under-representation of repeats observed here.

Another observation is consistent with the idea that the observed pattern is a consequence of selection: the bias against repeats is more pronounced in coding than in non-coding regions, as would be expected if it resulted from selection against targets of frame-shift mutations. However, because of possible constraints resulting from regulatory and structural functions in intergenic regions [30,31], this result has to be interpreted with caution. More direct evidence comes from the analysis of trinucleotide repeats. If a deletion bias would be responsible for the under-representation of repeats, one would expect this bias to also eliminate trinucleotide repeats. In contrast, if the under-representation would be a consequence of selection against targets of deleterious frame-shift mutations, one would expect selection to act much less efficiently towards eliminating trinucleotide repeats, because changes in them do not lead to a frame-shift. The missing evidence for a bias against trinucleotide repeats in the genome of *M. tuberculosis *might thus indicate that under-representation of mono-, di- and tetranucleotide repeats is a consequence of selection against targets for deleterious mutations. There was also evidence for the action of selection in a previous study with other organisms that investigated statistical associations between the occurrence of repeats and the expression level and essentiality of genes [22].

In this context, it is interesting to look at context-dependent codon choice in *Mycobacterium leprae*, a relative of *M. tuberculosis *that is also mismatch repair deficient. These two organisms differ in several important aspects, one of which is population size. As leprosy is much less common than tuberculosis, the effective population size of *M. leprae *is thought to be substantially smaller than the effective population size of *M. tuberculosis *[38]. In small populations, the efficiency of natural selection in removing deleterious mutations and promoting beneficial mutations is low [39]. As a consequence, one would expect that selection is less efficient in removing targets of frame-shift mutations. It is thus interesting to see that in *M. leprae*, context-dependent codon choice is generally weaker than in *M. tuberculosis*. The bias against nucleotide repeats is not as pronounced as in *M. tuberculosis*; in contrast to repeats of C and G, repeats of A and T are not significantly under-represented (Additional File 1), and the stabilizing effect of context-dependent codon choice is not as strong (Additional File 2). The weaker bias against repeats in *M. leprae *compared to *M. tuberculosis *is thus consistent with the interpretation that smaller populations in the former organism make selection for stability less efficient at removing nucleotide repeats from the genome.

However, the two types of explanations – selection and a neutral process – are not mutually exclusive, and they might both be involved in the observed pattern. And both explanations have the same final consequences: they lead to a genome that has fewer short nucleotide repeats and that is thus more stable. Interestingly, one would expect both types of processes to operate more strongly in organisms that lack an efficient mismatch repair system; in such organisms, selection for stable sequences would be stronger, and deletion biases would operate more quickly, thereby eliminating targets for frame-shift mutations. As a consequence, the lack of a mismatch repair system might, over evolutionary times, lead to genomes that partially compensate for this lack by an increased structural stability.

## Conclusion

In the present study we introduced the concept that a context-dependent codon choice can be an alternative to a mismatch repair system for attaining genetic stability. We showed that in the pathogenic, MMR-deficient *M. tuberculosis*, codons are used in a context-specific way that limits the emergence of short nucleotide repeats, and therefore reduces the number of targets for frameshift mutations. Context-dependent codon choice leads to a strong decrease in the estimated frame-shift mutation rate. This structural stability in *M. tuberculosis *is more pronounced than in the mismatch repair-proficient bacterium *E. coli*. This is consistent the idea that a deficiency in enzymatic DNA repair increases the selection for inherently stable genomes.

It is interesting to note that *M. tuberculosis *is an obligate pathogen, while *E. coli *is free-living. Genomes of bacterial pathogens often contain long mononucleotide repeats in genes involved in interactions with the host [14], and some genes of *M. tuberculosis *contain nucleotide repeats that show length polymorphisms between related genomes [27] and thus might play a role in the generation of functional variation. However, in general, context-dependent codon choice leads to a strong under-representation of short nucleotide repeats in *M. tuberculosis*. This is in line with the idea that all organisms have a core set of genes that are under selection for fulfilling their function in a stable and ordered manner [35], and suggests that in *M. tuberculosis *a dominant fraction of genes belongs to this group.

## Methods

### Origin of data

Sequence data of the whole genome and intergenic regions of *M. tuberculosis *H37Rv (genebank number AL123456) were retrieved from The J. Craig Venter Institute [40]. Sequence data of *E. coli *K12 were retrieved from NCBI [41] (accession no. 000913). Sequence data of *M. leprae *were retrieved from NCBI [41] (accession no. 002677).

### Comparing mononucleotide repeats in coding and intergenic regions with a null-model that assumes random distribution of nucleotides

To compare the distribution of mononucleotide repeats in coding and intergenic regions, we used a null-model based on a random distribution of nucleotides within each compartment. We first determined the observed distribution of mononucleotide repeats for each nucleotide, as well as the frequency of each nucleotide in both compartments. Then we calculated the expected distribution of mononucleotide repeats in both compartments with a permutation model. For example: the expected number of G_6 _repeats is determined by the probability of finding exactly 6 consecutive Gs by chance. As long as a compartment is reasonably large, this is very closely approximated by ((N-G)^2^/N^2^) × (G^6^/N^6^)N, where G is the number of Gs and N is the total number of all nucleotides within this compartment.

For each combination of nucleotide and repeat length, we then tested whether the proportions of expected and observed numbers differed between coding and intergenic regions. Observed and expected numbers in coding and intergenic regions were regarded as entries in a 2*2 contingency table, and a chi-square test was used to test for an association between the values for observed/expected and coding/noncoding.

### Comparing observed and expected numbers of mononucleotide repeats in coding regions with a null-model that preserves the amino acid sequence and the codon frequencies

The expected distribution of mononucleotide repeats in coding regions was determined with a null-model that preserved the amino acid sequence and the ORF-specific codon frequencies. For this analysis, we wrote programs in PERL. For each ORF, we first determined the codon frequencies and the amino acid sequence. Then, we generated a random nucleotide sequence that encoded the same amino acid sequence. For this randomized sequence, codons were drawn according to their frequencies in that particular ORF, but with replacement; the randomized sequence thus typically differed slightly from the actual ORF sequence in the codon frequencies. Next, we determined the number and length of all mononucleotide repeats of length > 1 in the randomized ORF sequence. We repeated these steps for each ORF in the genome, and summed up the number and length of all mononucleotide repeats over all ORFs. This resulted in one instance of the distribution of mononucleotide repeats in the genome that would be expected if codons were used in a context-independent manner. The whole procedure was repeated 100 times, so that we obtained 100 instances of the expected distribution of mononucleotide repeats under our null-model. From these 100 instances, we calculated the average expected number of repeats, as well as the distribution around this average, for every combination of nucleotide and length. We also determined the percentile range of 2.5–97.5 of this distribution.

If the observed number of mononucleotide repeats fell outside this range, we concluded that this observation was not consistent with the null-model; this indicated that repeats of this nucleotide and this particular length were significantly over- or underrepresented. For Fig. 2, the observed number of repeats as well as the 2.5–97.5 percentile range was standardized by dividing by the average of the expected number (for details see [22]). For practical reasons, we used randomization rather than exact calculation for determining the expected number of repeats under our modified null-model. In principle, one could also use exact calculation by determining all possible nucleotide sequences that encode a given amino acid sequence, calculating the probability of each nucleotide sequence under the assumption that codons are used independently of the sequence context, and then summing up the number of repeats in all nucleotide sequences after weighing for the probability of each sequence. However, even for short amino acid sequence, the number of possible nucleotide sequences is very high, and this approach is therefore not feasible.

### Determining the context-dependent usage of homogenous codons

In all ORFs in the genome of *M. tuberculosis *we used a program written in PERL to determine the occurrences of the four amino acids that can be encoded by a homogeneous codon (i.e., a codon that consists of three identical nucleotides) as well as one or more non-homogeneous codons (codons that do not consist of three identical nucleotides). These are phenylalanine (TTT and TTC), proline (CCA, CCC, CCG, and CCT), lysine (AAA and AAC), and glycine (GGA, GGC, GGG, and GGT). For each occurrence of one of these amino acids, we determined whether the codon was homogeneous or non-homogeneous, and whether it was followed by one or more of the nucleotides that constitute the homogeneous codon (for example, for each occurrence of a proline, we asked whether the codon used to encode the proline was CCC or any of CCA, CCG or CCT, and determined the number of C immediately following the codon). This data was used to determine how the usage of homogeneous codons changed at positions immediately followed by one or more nucleotides of the same type (Figure 3). The frequency of using the homogenous codons was normalized, taking into account their frequency in the genome. We used logistic regression to test whether the probability for a homogeneous codon changed with the number of nucleotides of the same type following. The program Jmp (version 6, SAS Institute Inc.) was used for logistic regression.

### Estimating the frameshift mutation rate

To estimate the frameshift mutation rates in mononucleotide repeats composed of different nucleotides and of different length, we used estimates of biological parameters from the literature. The first assumption was that the frame-shift mutation rate in a C_6 _repeat in Mycobacteria is about 3*10^7 ^[23]. The second assumption was that in bacteria, the frame-shift mutation rate in repeats of C and G is about five times higher than in repeats of A and T of the same length [42]. The third assumption was that in MMR deficient bacteria, the mutation rate in mononucleotide repeats increases by about a factor of three with each additional nucleotide that is added to the repeat [43]. A further assumption was that the frame-shift mutation rate in mononucleotide 'repeats' of length two and three (for example, CC and CCC) is negligible, and that only repeats of length four and longer have a significant frame-shift mutation rate.

With these assumptions, we could estimate the frame-shift mutation rate of individual mononucleotide repeats, and we could sum up the estimated frame-shift mutation rates over all repeats in a genome to get an estimate of the genome-wide frame-shift mutation rate. These estimates were particularly sensitive to the last two assumptions. For this reason, we varied these assumptions to obtain estimates of the frame-shift mutation rate for a whole range of parameter values (Figure 4). This lead to the following equations for estimated frame-shift mutation rates U_FS(n) _in a mononucleotide repeat of length *n *≥ *i *(where *i *is the minimal length that has a significant frame-shift mutation rate; mononucleotide repeats with a length smaller than *i *were assumed to have a frame-shift mutation rate of zero):

U_FS(n) _= 3*10^-7^/b*c^*n*-6^

where *b*, a parameter that accounts for differences in the mutation rate between repeats of A and T versus repeats of C and G, is equal to five for the nucleotides A and T and equal to one for C and G, and *c *is the factor by which the mutation rate increases with each additional nucleotide. The factor 3*10^-7 ^is based on the estimated frame-shift mutation rate in C_6 _repeats of Mycobacteria [23]. For example, the estimated frame-shift mutation rate of a A8 repeat is 3*10^-7^/5*3^2 ^= 5.4 * 10^-7 ^(for c = 3). This equation was used to calculate the estimated frame-shift mutation rate for every mononucleotide repeat in the real genome. We then added those rates to obtain the estimate of the total frame-shift mutation rate per genome. We used the same method to determine the estimated frame-shift mutation rate in the randomized genomes. To do so, we determined the average number of mononucleotide repeats (for every combination of nucleotide and length) in 100 randomized genomes, and calculated the estimated frame-shift mutation rate based on these averages. Based on published data (see above), we concluded that the parameter values *c *= 3 and *i *= 4 are most plausible. For Figure 4, the parameter *c *was varied from 2 to 4, and the parameter *i *was varied from 4 to 6.

### Comparing observed and expected numbers of di-, tri- and tetranucleotide repeats in coding regions with a null-model that preserves the amino acid sequence and the codon frequencies

To determine expected frequencies of di-, tri- and tetranucleotide repeats in the genome of *M. tuberculosis*, we used the same null-model as for mononucleotide repeats. We generated 100 random nucleotide sequences of the coding regions that preserved the amino acid sequence of each ORF and the ORF-specific codon frequencies. In these randomized sequences, the number and length of all di-, tri- and tetranucleotide repeats was determined (with modified versions of the PERL program used for mononucleotide repats). This data was used to calculate the average expected number of repeats, as well as the percentile range of 2.5–97.5, for every combination of nucleotide and length. These expected values were compared to the actual frequencies of these repeats in the genome of *M. tuberculosis*.

## Authors' contributions

All authors conceived of the study. MA and RMW performed the analysis. MA and RMW wrote the manuscript. ECB and BS edited and improved the manuscript.

## Supplementary Material

Additional file 1 Tables with observed and expected numbers of mono-, di-, tri- and tetranucleotide repeats in protein-coding genes in the genome of *M. tuberculosis*, and observed and expected numbers of mononucleotide repeats in protein-coding genes in the genomes of *M. leprae *and *E. coli*. Expected numbers of repeats are calculated with a null-model that preserves the amino acid sequence and the gene-specific codon frequencies. Click here for file

Additional file 2 Tables with estimated frame-shift mutation rates in mononucleotide repeats in protein coding genes of *M. tuberculosis*, *M. leprae *and *E. coli*. Estimates are given for the real genomes as well as for randomized genomes that preserve the amino acid sequence and the gene-specific codon frequencies. Click here for file
