# Supplementary material for: Stabilization of the genome of the mismatch repair deficient Mycobacterium tuberculosis by context-dependent codon choice
Source: BMC Genomics. 2008 May 28;9:249. doi: 10.1186/1471-2164-9-249 (PMC2430213; doi:10.1186/1471-2164-9-249)
Supplement: Additional file 1 — Tables with observed and expected numbers of mono-, di-, tri- and tetranucleotide repeats in protein-coding genes in the genome of M. tuberculosis, and observed and expected numbers of mononucleotide repeats in protein-coding genes in the genomes of M. leprae and E. coli. Expected numbers of repeats are calculated with a null-model that preserves the amino acid sequence and the gene-specific codon frequencies. [file 1471-2164-9-249-S1.pdf]

Additional File 1

1A) Observed and Expected Number of Mononucleotide Repeats (with 2.5 and 97.5 Percentile from 100 Randomizations) in *M. tuberculosis*

	A				C				G				T			
Len	Observed	Expected	2.5 PCTL	97.5 PCTL	Observed	Expected	2.5 PCTL	97.5 PCTL	Observed	Expected	2.5 PCTL	97.5 PCTL	Observed	Expected	2.5 PCTL	97.5 PCTL
1	450467	447866.12	447348	448379.5	666453	625663.88	624920.6	626594.3	631651	614709.88	613680.8	615404	471127	473546.87	472824.2	474226.9
2	88391	88075.42	87770.1	88362.5	223796	218346.67	217667.2	218967.4	250373	244639.25	244122.8	245226.1	84794	84416.62	84016.4	84831.9
3	12267	12887.92	12736.6	13059.8	45830	52459.53	52166.4	52685.9	52981	55785.88	55435.8	56128.3	13835	13487.83	13315.1	13677
4	2281	2483.74	2405.4	2554.7	8731	12830.87	12650	13014.1	11734	12508.02	12363	12682.7	2470	2343.41	2250.9	2438.5
5	402	455.16	411.8	488.6	1697	3558.91	3443.4	3664	2387	4353.19	4252.4	4461	584	501.56	457.5	545
6	44	86.69	72	101.5	233	842.53	796.9	887.6	374	1105.07	1060	1157	64	104.83	88	122
7	7	15.85	8	24	37	235.99	202	268	66	303.04	267.5	342.6	9	19.14	11	27.5
8	1	4.41	1	8.5	2	75.45	60.5	91.5	2	93.36	74	109.5	0	3.26	0.5	7
9	0	1.29	0	4	1	20.91	14	28.5	0	20.44	12	30	0	0.72	0	2.5
10	0	0.14	0	1	0	8.91	4	15	0	5.44	2	11	0	0.11	0	1
11	0	0.08	0	1	0	3.68	1	7.5	0	2.5	0	6	0	0.02	0	0
12	0	0.01	0	0	0	1.39	0	4	0	0.72	0	2	0	0	0	0
13	0	0	0	0	0	0.6	0	2	0	0.12	0	1	0	0	0	0
14	0	0	0	0	0	0.3	0	2	0	0.16	0	2	0	0.01	0	0
15	0	0	0	0	0	0.11	0	1	0	0.1	0	1	0	0	0	0
16	0	0	0	0	0	0.04	0	1	0	0.01	0	0	0	0	0	0
17	0	0	0	0	0	0.04	0	0.5	0	0.01	0	0	0	0	0	0
18	0	0	0	0	0	0	0	0	0	0	0	0	0	0	0	0

Abbreviations: Len: Length; PCTL: Percentile

1B) Observed and Expected Number of Dinucleotide Repeats (with 2.5 and 97.5 Percentile from 100 Randomizations) in *M. tuberculosis*

		Second Nuc.															
		A				C				G				T			
Len	First Nuc	Observed	Expected	2.5 PCTL	97.5 PCTL	Observed	Expected	2.5 PCTL	97.5 PCTL	Observed	Expected	2.5 PCTL	97.5 PCTL	Observed	Expected	2.5 PCTL	97.5 PCTL
1	A	.	.	.	.	160569	163714.35	163369.3	164200.1	101081	104120.9	103692	104478.5	128358	121227.54	120896	121591.2
2	A	.	.	.	.	9239	7957.97	7841	8064	3093	4052.47	3947.8	4177.8	2782	2952.77	2874.1	3030.5
3	A	.	.	.	.	315	234.86	207.5	266	55	108.39	87	129.5	48	49.68	38	61.5
4	A	.	.	.	.	8	8.95	3.5	14	4	4.51	1	10	3	0.9	0	3
5	A	.	.	.	.	2	0.59	0	2	0	0.19	0	1.5	0	0	0	0
6	A	.	.	.	.	0	0.03	0	0.5	0	0	0	0	0	0	0	0
7	A	.	.	.	.	0	0	0	0	0	0	0	0	0	0	0	0
8	A	.	.	.	.	0	0	0	0	0	0	0	0	0	0	0	0
1	C	187333	172139.42	171711.6	172568	.	.	.	.	325044	303102.43	302418.9	303799	163057	168109.74	167592.4	168716.4
2	C	7619	6782.36	6635.2	6910.7	.	.	.	.	31631	32078.69	31841.5	32362.5	3636	5017.4	4866.4	5130.6
3	C	318	249.64	219.5	277	.	.	.	.	2190	2609.03	2513.1	2696.1	77	151.22	124.3	177.1
4	C	13	11.85	6	17.5	.	.	.	.	206	302.11	271.5	332.2	1	5.05	1	10
5	C	1	0.77	0	3	.	.	.	.	25	38.98	26.5	48.5	0	0.24	0	1.5
6	C	0	0.11	0	1	.	.	.	.	0	3.13	0	7	0	0	0	0
7	C	0	0	0	0	.	.	.	.	0	0.54	0	2	0	0	0	0
8	C	0	0	0	0	.	.	.	.	0	0.07	0	1	0	0	0	0
1	G	217675	220446.89	220033.1	220909.8	275654	263524.08	262871.5	264041	.	.	.	.	178215	177714.15	177156.5	178228
2	G	8441	9360.75	9205.3	9525.1	31140	28819.65	28540.1	29058.4	.	.	.	.	11174	9850.34	9714.8	10038.7
3	G	89	209.48	184.5	236.5	3129	2902.57	2801.8	3005.1	.	.	.	.	377	359.96	334	398.1
4	G	1	6.81	2	13.5	277	310.91	276	344.2	.	.	.	.	22	16.91	11	24.5
5	G	0	0.29	0	1.5	13	34.67	25	44	.	.	.	.	0	1.3	0	4
6	G	0	0.06	0	1	0	3.57	0	8	.	.	.	.	0	0.03	0	0.5
7	G	0	0.02	0	0	0	0.49	0	2	.	.	.	.	0	0	0	0
8	G	0	0	0	0	0	0.04	0	1	.	.	.	.	0	0	0	0
1	T	52973	59946.27	59587.2	60265.5	185932	174625.35	174110.1	175139.3	176784	176128.23	175694.9	176565.1	.	.	.	.
2	T	854	1561.69	1481.3	1626.6	5924	6887.58	6770.9	7010.2	7174	7474.05	7350.2	7588.3	.	.	.	.
3	T	7	26.86	18	35	98	165.02	137.5	190	292	264.08	233.4	291.1	.	.	.	.

4	T	0	0.45	0	2	0	6.06	2	12	22	17.87	11	24.5	.	.	.	.
5	T	0	0	0	0	0	0.2	0	1	0	0.64	0	2.5	.	.	.	.
6	T	0	0	0	0	0	0.01	0	0	0	0	0	0	.	.	.	.
7	T	0	0	0	0	0	0	0	0	0	0	0	0	.	.	.	.
8	T	0	0	0	0	0	0	0	0	0	0	0	0	.	.	.	.

Abbreviations and Notes: Nuc: Nucleotide; Len: Length; PCTL: Percentile. ‘First Nuc.’ and ‘Second Nuc. refer to the first and the second nucletide in a dinucledotide. The dinucleotides AA, CC, GG and TT were not listed, because they also represent mononucleotide repeats of length two.

1C) Observed and Expected Number of Trinucleotide Repeats (with 2.5 and 97.5 Percentile from 100 Randomizations) in *M. tuberculosis* (one GCC_7 repeat is not listed; there are no repeats of length 6).

	Length 1				2				3				4				5			
Tri-nucleotide	Observed	Expected	2.5 PCTL	97.5 PCTL	Obs	Exp	2.5 PC.	97.5 PC.	Obs	Exp	2.5 PC.	97.5 PC.	Obs	Exp	2.5 PC.	97.5 PC.	Obs	Exp	2.5 PC.	97.5 PC.
AAC	23086	27574	27374	27832	336	438	411	466	11	18	11	27	1	0.2	0.0	1.0	0	0.0	0.0	0.0
AAG	22834	20916	20730	21117	429	359	336	390	11	8	3	13	2	0.8	0.0	2.0	0	0.0	0.0	0.0
AAT	18497	18160	17951	18329	29	56	44	68	0	1	0	3	0	0.0	0.0	0.0	0	0.0	0.0	0.0
ACA	27360	24676	24494	24863	633	465	433	493	17	12	8	17	0	0.0	0.0	0.0	0	0.0	0.0	0.0
ACC	52762	57718	57445	58041	1419	1550	1483	1619	56	69	55	81	4	2.8	1.0	6.0	0	0.2	0.0	1.0
ACG	45226	45296	45074	45604	1725	1439	1389	1479	99	67	55	81	6	4.0	2.0	7.0	0	0.1	0.0	1.0
ACT	26329	26243	25972	26526	358	337	310	368	5	5	3	8	0	0.0	0.0	0.0	0	0.0	0.0	0.0
AGA	18271	21585	21408	21769	324	346	320	375	12	8	5	13	0	0.2	0.0	1.0	0	0.0	0.0	0.0
AGC	34778	39226	38937	39486	782	877	837	928	17	25	18	32	1	1.0	0.0	3.0	0	0.0	0.0	0.0
AGG	27982	28519	28340	28711	375	502	465	535	5	10	5	15	1	0.3	0.0	1.0	0	0.0	0.0	0.0
AGT	22104	24896	24667	25097	42	75	58	94	0	1	0	3	0	0.0	0.0	0.0	0	0.0	0.0	0.0
ATA	7816	10148	9962	10324	9	39	28	51	0	0	0	1	0	0.0	0.0	0.0	0	0.0	0.0	0.0
ATC	43375	39144	38906	39418	889	787	753	827	28	18	13	25	1	0.6	0.0	1.0	0	0.0	0.0	0.0
ATG	25104	27076	26885	27305	282	423	397	447	14	15	11	19	2	1.9	1.0	3.0	0	0.0	0.0	0.0
ATT	19727	17725	17539	17948	76	99	79	118	0	1	0	3	0	0.0	0.0	0.0	0	0.0	0.0	0.0
CAA	45348	36725	36470	37032	883	614	582	649	51	29	22	39	0	0.3	0.0	1.0	0	0.0	0.0	0.0
CAC	47885	39959	39725	40226	1516	1112	1063	1175	60	49	38	61	3	1.4	0.0	4.5	0	0.7	0.0	2.0
CAG	35071	34984	34684	35269	928	884	849	938	26	28	20	37	0	0.6	0.0	2.0	0	0.2	0.0	1.0
CAT	35244	35504	35270	35768	812	755	715	789	23	21	16	27	0	0.0	0.0	0.0	0	0.0	0.0	0.0
CCA	48615	49608	49240	50003	1462	1298	1233	1357	50	45	34	60	3	2.3	0.0	4.5	0	0.0	0.0	1.0
CCG	88234	87649	87302	88026	3971	3882	3792	3971	270	264	238	290	14	23.6	15.5	31.5	1	3.4	1.0	6.5
CCT	39674	52448	52148	52751	246	811	752	865	2	13	6	20	0	0.2	0.0	1.0	0	0.0	0.0	0.0
CGA	93347	79389	78989	79880	4822	2965	2882	3046	265	146	129	167	15	8.3	4.5	13.0	0	0.1	0.0	1.0
CGC	86260	82726	82351	83108	4706	4366	4252	4472	311	312	284	339	19	29.8	20.5	39.5	0	4.4	2.0	8.0
CGG	105490	99631	99208	100044	8596	6466	6302	6576	391	267	235	295	36	27.3	19.5	35.5	14	7.9	3.0	13.5
CGT	52673	58196	57872	58547	1706	1715	1651	1782	86	72	54	88	5	3.4	0.5	7.5	1	0.1	0.0	1.0
CTA	20675	19402	19195	19640	275	222	197	244	2	3	1	6	0	0.0	0.0	0.0	0	0.0	0.0	0.0
CTC	32947	31987	31730	32240	230	452	416	487	3	6	2	12	0	0.1	0.0	1.0	0	0.0	0.0	0.0
CTG	44381	53389	53032	53699	2131	2281	2204	2352	110	103	87	120	4	5.8	2.0	10.0	0	0.1	0.0	1.0
CTT	23073	28850	28630	29112	427	577	543	621	8	10	5	15	0	0.1	0.0	1.0	0	0.0	0.0	0.5

GAA	35281	41143	40852	41396	541	643	606	684	16	12	6	18	1	0.7	0.0	2.0	0	0.0	0.0	0.0
GAC	43920	53094	52855	53400	1259	1722	1667	1771	70	78	63	94	4	5.0	2.0	9.0	0	0.0	0.0	0.0
GAG	38456	37128	36810	37417	985	829	784	870	23	21	13	28	0	0.1	0.0	1.0	0	0.0	0.0	0.0
GAT	48634	42309	41951	42612	737	736	689	773	12	17	11	24	0	0.4	0.0	2.0	0	0.0	0.0	0.0
GCA	66322	58126	57740	58408	1372	1278	1224	1341	40	38	28	50	0	0.7	0.0	2.0	0	0.1	0.0	1.0
GCC	81920	83121	82676	83503	4604	4522	4405	4641	321	335	298	379	19	31.8	22.0	40.5	2	3.9	1.0	8.0
GCG	77860	77306	76978	77774	4046	3818	3718	3911	265	262	235	289	21	18.3	11.0	27.5	6	2.3	0.0	5.0
GCT	63031	57199	56901	57452	2691	1810	1741	1884	128	70	57	86	11	4.1	1.0	8.0	0	0.1	0.0	1.0
GGA	50061	67203	66814	67592	588	1234	1179	1285	8	26	18	34	0	0.2	0.0	1.0	0	0.0	0.0	0.0
GGC	83466	88081	87716	88445	4648	5120	4984	5252	305	305	274	334	22	29.6	20.5	41.5	3	6.0	2.0	10.0
GGT	67257	58033	57747	58355	2867	2002	1930	2093	141	89	73	103	5	4.0	1.0	9.0	1	0.2	0.0	1.0
GTA	16601	21611	21375	21865	28	78	60	97	0	1	0	3	0	0.0	0.0	0.0	0	0.0	0.0	0.0
GTC	45368	45169	44901	45499	1197	1272	1219	1338	68	49	36	62	4	2.0	0.0	5.0	0	0.0	0.0	1.0
GTG	37178	44913	44592	45201	1909	1761	1702	1813	82	88	71	106	5	4.1	0.5	7.0	2	0.3	0.0	1.5
GTT	36676	32154	31864	32471	709	479	441	514	24	9	3	15	0	0.2	0.0	1.0	0	0.0	0.0	0.0
TAA	5320	8284	8121	8464	13	32	21	43	0	1	0	3	0	0.0	0.0	0.0	0	0.0	0.0	0.0
TAC	16493	20350	20129	20540	241	256	233	275	2	2	0	4	0	0.0	0.0	0.0	0	0.0	0.0	0.0
TAG	6177	8085	7938	8242	23	38	27	51	0	0	0	2	0	0.0	0.0	0.0	0	0.0	0.0	0.0
TAT	14235	15200	14969	15406	17	82	66	100	0	0	0	2	0	0.0	0.0	0.0	0	0.0	0.0	0.0
TCA	33660	27987	27734	28248	728	688	650	737	24	14	7	19	0	0.0	0.0	0.5	0	0.0	0.0	0.0
TCC	40013	42589	42329	42840	174	569	522	618	0	9	4	14	0	0.2	0.0	1.5	0	0.0	0.0	0.0
TCG	71973	67967	67607	68358	2739	1920	1850	2005	118	68	56	80	10	3.2	1.0	6.5	0	0.1	0.0	1.0
TCT	20989	23936	23689	24201	236	330	298	358	11	11	7	16	0	0.1	0.0	1.0	0	0.0	0.0	0.0
TGA	37961	39757	39490	40006	738	641	602	679	10	12	7	18	0	0.1	0.0	1.0	1	0.8	0.0	1.0
TGC	52518	45483	45209	45760	2322	1580	1515	1638	127	73	61	88	4	3.9	0.5	7.0	0	0.2	0.0	1.0
TGG	65532	68537	68272	68820	2513	2629	2565	2698	126	108	92	127	8	7.2	3.5	12.5	0	0.6	0.0	2.0
TGT	30550	29477	29280	29702	619	427	386	469	14	7	3	13	0	0.2	0.0	1.0	0	0.0	0.0	0.0
TTA	6749	9087	8923	9294	22	50	37	63	0	0	0	2	0	0.0	0.0	0.0	0	0.0	0.0	0.0
TTC	35558	28622	28449	28804	692	538	504	566	21	14	9	19	0	0.1	0.0	1.0	0	0.1	0.0	1.0
TTG	23572	27516	27248	27813	344	411	373	446	9	7	2	13	0	0.2	0.0	1.0	0	0.0	0.0	0.0

Abbreviations and Notes: Nuc: Nucleotide; Len: Length; Obs: Observed; Exp: Expected; PC: Percentile. The trinucleotides AAA, CCC, GGG and TTT were not listed, because they also represent mononucleotide repeats of length three.

1D) Observed and Expected Number of Tetranucleotide Repeats (with 2.5 and 97.5 Percentile from 100 Randomizations) in *M. tuberculosis*

	Length 1				2				3				4			
Tetra-nucleotide	Observed	Expected	2.5 PCTL	97.5 PCTL	Observed	Expected	2.5 PCTL	97.5 PCTL	Observed	Expected	2.5 PCTL	97.5 PCTL	Observed	Expected	2.5 PCTL	97.5 PCTL
AAAG	4926	5075	4948.2	5185.7	8	6.3	1.5	11.5	0	0	0	0	0	0	0	0
AAAC	6014	6086	5982.4	6190.5	6	8.4	3.5	14	0	0	0	1	0	0	0	0
AAAT	3449	4124.4	4015.4	4232.5	0	1.2	0	4	0	0	0	0	0	0	0	0
AACA	8445	8263.4	8172.9	8351.7	11	7.7	3	14	0	0	0	1	0	0	0	0
AACC	11395	11751.5	11628.5	11932.7	46	36.5	27	48	0	0.1	0	1	0	0	0	0
AACG	15059	14069.5	13946	14231.7	29	36.9	28.5	45.5	0	0.1	0	1	0	0	0	0
AACT	8010	8106.1	7965.8	8236.6	3	2.1	0	5	0	0	0	0	0	0	0	0
AAGA	7574	7165.9	7070	7287.5	14	9.8	5	14	0	0	0	0	0	0	0	0
AAGC	9858	10762.5	10633.9	10920.4	19	30.9	21.5	41	0	0.1	0	1	0	0	0	0
AAGG	10282	9637	9514.6	9723.1	26	21	14.5	29	0	0	0	0	0	0	0	0
AAGT	5250	6249.5	6129.3	6342.5	0	1.2	0	3.5	0	0	0	0	0	0	0	0
AATA	2387	2550.6	2478.9	2628.2	0	1.8	0	4.5	0	0	0	0	0	0	0	0
AATC	7014	7074.1	6937	7206.5	11	12.5	7	19	0	0	0	1	0	0	0	0
AATG	5466	5483.6	5379.4	5598.5	3	4.8	1.5	8.5	0	0	0	0	0	0	0	0
AATT	3991	4065.9	3963.6	4150.6	1	2.2	0	5	0	0	0	0	0	0	0	0
ACAA	8538	7485	7383.5	7587.8	13	10.5	6	15	0	0	0	0	0	0	0	0
ACAG	7132	7855.8	7710.9	8018.4	13	13.6	5	20.5	0	0	0	0	0	0	0	0
ACAT	9229	8404	8311	8502.6	1	4.7	1	9	0	0	0	0	0	0	0	0
ACCA	16110	15295.4	15171.2	15420.6	57	52.3	44	63.1	0	0.1	0	1	0	0	0	0
ACCC	16883	19470.6	19288.4	19638.4	84	80.6	67	97.5	0	0.3	0	2	0	0	0	0
ACCG	34248	29466.1	29194.2	29697.3	251	205.6	183	226.1	0	0.9	0	3	0	0	0	0
ACCT	14155	14959.7	14763.4	15101.5	73	56.8	44.4	68.1	0	0	0	0.5	0	0	0	0
ACGA	18145	16827.6	16674.3	16964.1	28	46.4	35.5	58	0	0.1	0	1	0	0	0	0
ACGC	22157	21561.3	21378.6	21749.1	65	67.8	54	81.5	0	0.3	0	1.5	0	0	0	0
ACGG	23130	22455.6	22172.9	22664.2	52	96.5	76.5	112.5	0	0.4	0	2	0	0	0	0
ACGT	13491	13050.1	12910.4	13187.3	9	26.1	19	35.5	0	0	0	1	0	0	0	0
ACTA	4662	4595	4477.1	4678.1	0	2.5	0	5	0	0	0	0	0	0	0	0
ACTC	7788	7293	7153.9	7425.4	3	11.3	5	17.5	0	0	0	0	0	0	0	0
ACTG	11344	11135.3	10996.8	11295.6	12	14.3	9	21	0	0	0	0	0	0	0	0
ACTT	6447	7257.3	7135.4	7379.2	6	5.1	2	10.5	0	0	0	0	0	0	0	0

AGAA	5515	5947.8	5851.5	6066.2	6	7.4	3	12.5	0	0	0	0	0	0	0	0
AGAC	8467	9530	9408.4	9650.6	13	14.9	9	24.5	0	0	0	1	0	0	0	0
AGAT	8526	8204.8	8088	8304	0	4.5	1	8.5	0	0	0	0	0	0	0	0
AGCA	10690	10691.1	10576	10846.6	29	31	20.5	42.5	0	0	0	1	0	0	0	0
AGCC	14018	15334.2	15165.3	15504.8	80	66.8	50.4	81	0	0.1	0	1	0	0	0	0
AGCG	17928	18635.9	18437.3	18839.2	92	89.4	72.4	107.5	0	0.3	0	1.5	0	0	0	0
AGCT	13004	13076.5	12910.4	13222.3	10	8.4	4	16	0	0	0	0	0	0	0	0
AGGA	8364	8475.9	8363	8586.2	13	11.8	7	18.1	0	0	0	0	0	0	0	0
AGGC	14048	14980.7	14772	15172.4	58	59.2	44.5	73.5	0	0.1	0	1	0	0	0	0
AGGG	7780	8260.1	8143.2	8370.1	8	17.8	9	25	0	0	0	0	0	0	0	0
AGGT	11223	10562.4	10430.3	10698.3	9	8.1	3	13	0	0	0	0	0	0	0	0
AGTA	2758	3430.5	3349.4	3516.1	0	1.9	0	5.5	0	0	0	0	0	0	0	0
AGTC	6537	7692.3	7553.3	7811.1	15	14.8	7	25	0	0	0	0	0	0	0	0
AGTG	6924	8261.6	8128.9	8432.1	14	14.5	8.5	22	0	0	0	0	0	0	0	0
AGTT	6640	6794.9	6674.7	6912.5	2	2.2	0	6.5	0	0	0	0	0	0	0	0
ATAA	1129	2279.3	2213.3	2346.3	0	0.9	0	3	0	0	0	0	0	0	0	0
ATAC	4193	4568.4	4457.9	4667.7	7	5	1	9	0	0	0	0	0	0	0	0
ATAG	1601	2372.2	2268.5	2464.1	0	1.3	0	4	0	0	0	0	0	0	0	0
ATCA	12357	10846.4	10757.5	10943.4	16	16.5	10.5	22.5	0	0	0	0	0	0	0	0
ATCC	13930	12717.2	12602.4	12840.4	31	30.2	21	38	0	0.1	0	1	0	0	0	0
ATCG	26016	24588.3	24428.8	24740.8	113	100.5	79.5	123.2	0	0.2	0	1	0	0	0	0
ATCT	9318	8687.4	8561.6	8834.9	36	25.5	18	33.5	0	0	0	0	0	0	0	0
ATGA	8540	9347.9	9227.7	9472.4	8	14.9	8	21	0	0	0	0	0	0	0	0
ATGC	12651	11758.4	11599.4	11912.6	25	23.2	16.5	30.5	0	0	0	0	0	0	0	0
ATGG	13019	13772.1	13648.3	13920.1	30	33.8	24.5	43.5	0	0.1	0	1	0	0	0	0
ATGT	7906	7267.2	7163.2	7394.5	6	5.1	1	9	0	0	0	0	0	0	0	0
ATTA	1870	2776.8	2696	2865.3	1	1.5	0	4	0	0	0	0	0	0	0	0
ATTC	8567	5981.5	5854.9	6114.2	15	7.6	2	12.5	0	0	0	0	0	0	0	0
ATTG	7184	6977.7	6827	7141.4	12	11.7	7	17.5	0	0	0	0	0	0	0	0
ATTT	4315	4024.6	3908.2	4127.1	4	4.3	1	9.5	0	0	0	0	0	0	0	0
CAAA	6879	6394.2	6269	6531	11	8.8	4	14.5	0	0	0	0	0	0	0	0
CAAC	21693	16610.2	16449.8	16779.6	55	51.1	39.5	64	1	0.1	0	1	0	0	0	0
CAAG	15947	12726.6	12574.4	12885.2	38	34.8	25	46	0	0	0	0.5	0	0	0	0
CAAT	7241	6436	6313.9	6584.4	12	12.5	7	20	0	0	0	0	0	0	0	0
CACC	33570	26345.8	26131.4	26555.1	148	107.9	89.9	125	0	0.3	0	1	0	0	0	0
CACG	17477	17533.4	17288.8	17740.5	76	67.6	54.5	81.5	0	0.2	0	2	0	0	0	0

CACT	7957	7187.8	7042.7	7306.7	13	12.3	6.5	19.5	0	0	0	0	0	0	0	0
CAGA	8440	9793.5	9692	9906	10	14.6	7.5	23	0	0	0	0.5	0	0	0	0
CAGC	22398	20420.4	20220.3	20627.1	138	88.3	70.4	107.6	1	0.2	0	1	0	0	0	0.5
CAGG	14125	14690.7	14517.4	14824.1	34	47.3	35	60.5	0	0.2	0	1	0	0	0	0
CAGT	7838	8587.8	8410.2	8730.4	16	16.6	10.5	23	0	0	0	0	0	0	0	0
CATA	2357	3246.7	3163.4	3336.2	4	2.3	0	5	0	0	0	0	0	0	0	0
CATC	22930	21550.3	21333.4	21775.2	46	45.4	36.5	56	0	0	0	1	0	0	0	0
CATG	11631	12636.8	12499.4	12791.9	26	25.7	18	36.6	0	0.1	0	1	0	0	0	0
CATT	6694	6046.1	5916.3	6154.5	14	8.3	4	13.5	0	0	0	0	0	0	0	0
CCAA	15629	13012.1	12851.5	13188.2	37	39.9	30	53	0	0.1	0	1	0	0	0	0
CCAC	19295	19100.8	18953.7	19344.2	128	109.5	92.5	128	0	0.2	0	2	0	0	0	0
CCAG	17835	18204.4	17995.7	18413.9	116	84.3	66.9	101.5	1	0.3	0	2	0	0	0	0
CCAT	11554	13904.6	13726.2	14062.6	29	37.2	28	49	0	0.1	0	1	0	0	0	0
CCCA	12562	14594.9	14401.3	14773.7	98	77.2	62.5	90	0	0.4	0	1.5	0	0	0	0
CCCG	29464	32973.9	32733.4	33229	208	273.6	247.5	307.1	2	2.6	0	6	0	0.1	0	1
CCCT	8467	16002.9	15829.3	16190	3	27.7	19	40	0	0	0	1	0	0	0	0
CCGA	35790	27727.9	27497.9	27949.1	190	209.8	186.5	232.6	1	1.1	0	3	0	0.1	0	1
CCGC	43416	42564.7	42312	42829.5	358	438.5	399	474.2	2	3.3	0	7	0	0	0	0
CCGG	47245	44683.9	44360.2	44976	640	533.7	486	576.5	4	6.9	2	12	0	0.1	0	1
CCGT	19345	22841.8	22620.5	23061.8	45	92	75	108.1	0	0.3	0	1.5	0	0	0	0
CCTA	6557	6519.8	6385	6665	24	16.9	10.5	24.5	0	0	0	1	0	0	0	0
CCTC	10772	15265.2	15045.6	15525.2	4	37.9	28	50.5	0	0.1	0	1	0	0	0	0
CCTG	22893	31494.8	31220.4	31732.4	83	79.8	66	95.6	0	0.4	0	2	0	0	0	0
CCTT	6556	10382.2	10210	10555.5	10	18.2	11	24.5	0	0	0	0	0	0	0	0
CGAA	17468	17689.8	17484.4	17876.3	41	53.9	39	66.5	0	0.1	0	1	0	0	0	0
CGAC	40444	33690.5	33423.5	33983.8	193	238.8	207	270.6	1	0.9	0	3	0	0	0	0
CGAG	27399	24075	23874.9	24305.1	78	109.3	93.5	131.1	0	0.3	0	1	0	0	0	0
CGAT	24648	18813.4	18614.9	19025.7	135	110.5	92	128.6	1	0.2	0	1	0	0	0	0
CGCA	23948	21303.7	21032.2	21521.9	89	82.2	66.5	97.6	0	0.5	0	2	0	0	0	0
CGCC	47261	47263.3	47002.6	47537.6	347	411.8	383.5	446.1	3	3	0	6	0	0	0	0
CGCT	27053	22061.7	21833.2	22294.1	124	110.1	90.9	124.5	0	0.5	0	2	0	0	0	0
CGGA	19761	27343.8	27093.4	27614.1	54	134.7	116.4	154	0	0.5	0	2	0	0	0	0
CGGC	59806	52432.8	52110.5	52744.2	762	652.9	614.5	693	6	7.5	2.5	13	0	0.1	0	1
CGGG	32330	34940	34692	35186.4	327	360.3	325	401.5	0	2.5	0	5	0	0	0	0
CGGT	35939	28505.8	28262.9	28804.8	247	167.3	147	188.5	2	1	0	3	0	0	0	0
CGTA	4881	7618	7467.1	7780.6	9	15.9	9	22.5	0	0	0	0	0	0	0	0

CGTC	25334	25163.7	24923.9	25435.9	49	95.5	77.5	111.1	0	0.1	0	1	0	0	0	0
CGTG	21546	28362.6	28119.3	28663.4	85	105.3	87.9	126.1	0	0.4	0	1.5	0	0	0	0
CGTT	15258	12714.3	12519	12927.3	37	44.9	30	55	0	0.1	0	1	0	0	0	0
CTAA	1806	2547.4	2472.4	2640.5	1	1.3	0	3.5	0	0	0	0	0	0	0	0
CTAC	13441	11066.7	10874.1	11216.3	45	24.5	14.4	35	0	0	0	1	0	0	0	0
CTAG	2857	3459	3342.3	3572.4	0	2.7	0	5	0	0	0	1	0	0	0	0
CTAT	5584	5600.8	5477.7	5734.1	7	8	3	13.1	0	0	0	0	0	0	0	0
CTCA	9254	7459.3	7316.5	7601.7	11	11.1	5.5	17	0	0	0	0	0	0	0	0
CTCC	8333	12695.9	12516.3	12865.4	3	35.9	25.5	49.5	0	0.1	0	1	0	0	0	0
CTCG	20182	20017.1	19769.9	20223.5	88	87.5	71.5	103.5	0	0.4	0	2	0	0	0	0
CTGA	14689	15775	15577	15939.3	19	31.9	22.5	42.5	0	0	0	0	0	0	0	0
CTGC	25821	22384.3	22160.5	22629.8	60	59.2	44	73	0	0.4	0	1	0	0	0	0
CTGG	34452	35974.4	35700.5	36221.4	291	267.1	241.4	296.1	1	1.7	0	4	0	0.1	0	1
CTGT	10969	11505.3	11339.4	11685.6	17	17.8	9.5	25	1	0.1	0	1	0	0	0	0
CTTA	1859	2644.6	2544.8	2730.5	3	2.1	0	4.5	0	0	0	0	0	0	0	0
CTTC	12666	15097	14903.7	15275.2	5	18.1	11.5	24.5	0	0	0	0	0	0	0	0
CTTG	8619	12289.1	12109.3	12481.5	26	25.5	17.5	36	0	0	0	0.5	0	0	0	0
CTTT	4824	5228.9	5115.9	5337.1	9	5.3	2	10.5	0	0	0	0	0	0	0	0
GAAA	6808	7527.4	7372.1	7674.7	7	9.4	5	15.5	0	0	0	0	0	0	0	0
GAAC	13542	16346.7	16147.1	16499.5	37	51.4	41.5	62.1	0	0	0	0.5	0	0	0	0
GAAG	11792	14737.9	14556.9	14945.1	22	35.3	27	47.1	0	0	0	0	0	0	0	0
GAAT	7221	6964.7	6855	7108.1	4	7.6	3	13	0	0	0	0	0	0	0	0
GACA	14547	12874.1	12715.2	13050.1	11	20.4	11.5	29	0	0	0	0	0	0	0	0
GACC	25539	30145.6	29912.2	30364.6	190	234.3	206	264.1	0	0.7	0	2.5	0	0	0	0
GACG	32748	29271.2	29100.8	29509.3	61	158.3	138	181.6	1	0.8	0	3	0	0	0	0
GACT	10011	10524.8	10373.9	10651.7	13	13	5.5	20	0	0	0	0	0	0	0	0
GAGC	17847	20558.7	20361.4	20789.6	62	102	85	118.5	0	0.3	0	1	0	0	0	0
GAGG	16013	16590.4	16427.9	16759.6	9	32.5	20.5	42.5	0	0	0	0	0	0	0	0
GAGT	8130	9121.8	8976.9	9234.8	7	10	5	15.5	0	0	0	0	0	0	0	0
GATA	4519	5234.9	5115	5379.3	0	5.4	1	12	0	0	0	0	0	0	0	0
GATC	26988	22715.8	22533.1	22954.7	148	113.1	92.4	135.5	2	0.3	0	1	0	0	0	0
GATG	18982	16687	16503.3	16895.3	57	46.1	36	59.5	1	0.1	0	1	0	0	0	0
GATT	8632	7180.5	7018.2	7322.1	8	11	6	18.5	0	0	0	0	0	0	0	0
GCAA	17376	14323.2	14144.8	14519.4	42	36.2	26	47.1	0	0.1	0	1	0	0	0	0
GCAC	23174	18979.7	18808.4	19174.4	104	67	53.5	81	0	0.3	0	1.5	0	0	0	0
GCAG	21721	21753.9	21534.2	21971.5	60	85.3	73.4	103.5	2	0.2	0	1	0	0	0	0

GCAT	14484	13196.1	13009.4	13356.5	26	26.1	19.5	33.5	0	0	0	0.5	0	0	0	0
GCCA	25405	23613.3	23386.2	23809.5	133	98	82.5	112	4	0.4	0	2	0	0	0	0
GCCC	23481	29590.7	29354.9	29785.4	170	241.7	214.4	272.7	1	2.7	0	6.5	0	0.1	0	1
GCCG	59006	51583.6	51233.4	51938.8	668	623.4	566.8	664	8	6.6	2	11.5	0	0.2	0	1
GCCT	15191	20031.7	19815.8	20233.7	74	65.7	54.5	78.5	1	0.3	0	2	0	0	0	0
GCGA	29326	27290.7	27062.7	27564.5	92	125	105.5	148.5	0	0.3	0	2	0	0	0	0
GCGG	50817	50140.2	49780.7	50438.8	557	737	681.8	788.5	2	3.4	1	7	0	0	0	1
GCGT	19511	21346	21117.3	21560.5	61	81.2	69.5	96.5	0	0.2	0	1	0	0	0	0
GCTA	9528	8654.6	8526.3	8847.4	7	10.5	5	16.5	0	0	0	0	0	0	0	0
GCTC	18144	16720.1	16488.2	16931.6	69	74.8	60	92	0	0.4	0	1.5	0	0	0	0
GCTG	42920	33833.8	33633.5	34168.2	396	328.9	299	359.1	2	2.2	0	5	0	0.1	0	1
GCTT	9903	11139.8	10972.2	11339.2	14	20.5	13	28.5	0	0	0	1	0	0	0	0
GGAA	10521	13692.1	13529.6	13896	28	22.8	15.5	31.5	0	0	0	0	0	0	0	0
GGAC	18605	26046.5	25864.4	26307.1	50	110	90.5	130.5	0	0.7	0	3	0	0	0	0
GGAG	14121	20865.2	20659.4	21087.4	20	47.1	34	61	0	0.1	0	1	0	0	0	0
GGAT	14317	14229.3	14030	14419.6	50	39.5	30	49.5	0	0.1	0	1	0	0	0	0
GGCA	28951	24661.3	24437.1	24916.2	108	88.9	76.5	103.5	1	0.3	0	1.5	0	0	0	0
GGCC	41325	44002.8	43741.1	44291.2	507	537.9	496.5	575.6	5	5.7	2	11	2	0.1	0	1
GGCG	54151	50935.7	50609.8	51229	406	569.4	527.8	614.2	6	3.5	1	7	0	0.1	0	1
GGCT	22230	20503.7	20304.5	20709.5	256	227.8	206	246.5	0	1.8	0	5	0	0	0	0
GGGA	12582	16929.9	16723.9	17162	12	37.3	25.4	48.5	0	0	0	1	0	0	0	0
GGGC	29020	31965.4	31684.2	32219.2	302	303	278.5	332	6	2.8	0	6	0	0	0	1
GGGT	18795	17203.6	17006.6	17377.1	119	105.2	89	121	0	0.6	0	2	0	0	0	0
GGTA	8389	9524.1	9340	9702.2	7	9.9	5	16	0	0	0	0	0	0	0	0
GGTC	24334	23813.1	23550.4	24031.3	151	132.1	112	151.1	0	0.9	0	3	0	0	0	0
GGTG	40042	30721.9	30444.1	31013.3	232	206.1	178.5	231.1	1	0.6	0	2	0	0	0	0
GGTT	14506	11935.5	11740.9	12129.4	65	56.6	42.5	69	0	0.2	0	1	0	0	0	0
GTAA	2445	3612.3	3523.7	3715.5	3	1.6	0	4	0	0	0	0	0	0	0	0
GTAC	7843	10367.1	10211.9	10508.5	13	17.9	9.5	26.5	0	0	0	1	0	0	0	0
GTAG	3028	3997.7	3877	4101.2	3	3.1	0	6	0	0	0	0	0	0	0	0
GTAT	5306	6191.5	6032.3	6336.8	7	7.9	3	14	0	0	0	0	0	0	0	0
GTCA	14975	12345.3	12156.5	12521.4	24	21.8	14	28.5	0	0	0	0.5	0	0	0	0
GTCC	12327	16618.8	16406.1	16827.5	37	69.6	55.5	82.1	0	0.2	0	1	0	0	0	0
GTCG	31725	27514.5	27271.3	27756.1	194	166.4	143	188.5	0	0.7	0	3	0	0	0	0
GTCT	7322	9589.7	9455	9758.8	11	15	8.5	23	0	0.1	0	1	0	0	0	0
GTGA	15113	14883.7	14717.3	15072.9	14	23.6	15	32	0	0	0	0	0	0	0	0

GTGC	24748	19754.6	19585.6	19946.3	126	90.3	70.5	110	0	0.4	0	2	0	0	0	0
GTGG	26879	30889.6	30625.8	31151.1	176	192.2	166	219.2	0	0.5	0	2	0	0	0	0
GTTA	3175	3985.9	3855.5	4115.6	0	2.1	0	5	0	0	0	0	0	0	0	0
GTTC	17667	15256	15075.2	15448.6	48	47.1	33	65	0	0.1	0	1	0	0	0	0
GTTG	17819	14201.9	14020.9	14428.9	68	64	48.4	78.1	0	0.2	0	1	0	0	0	0
GTTT	7284	6618.9	6480	6783	4	11.1	6	17	0	0	0	0	0	0	0	0
TAAA	831	1477.7	1411.4	1540.6	0	0.6	0	2.5	0	0	0	0	0	0	0	0
TAAC	2399	3959.4	3868.7	4060.8	2	1.2	0	3	0	0	0	0	0	0	0	0
TAAG	1416	2467	2385.4	2556.2	0	0.8	0	3	0	0	0	0	0	0	0	0
TAAT	1051	1797	1733.5	1866.6	0	1	0	3.5	0	0	0	0	0	0	0	0
TACA	4951	4597.7	4498	4686.1	2	3.2	0	6	0	0	0	0	0	0	0	0
TACC	10059	10895.1	10735.5	11058.5	40	27.1	18	36.5	0	0	0	1	0	0	0	0
TACG	10129	10141	10000	10286.8	14	17.4	10	25	0	0	0	0	0	0	0	0
TACT	4357	4564.7	4466.4	4658.5	2	4	1	9	0	0	0	0	0	0	0	0
TAGA	1246	1961.8	1876.2	2054.1	1	0.6	0	2	0	0	0	0	0	0	0	0
TAGC	3277	4209	4085.4	4349.1	0	3.1	0.5	6	0	0	0	0	0	0	0	0
TAGG	1401	1889.8	1812.3	1962.6	0	1	0	3	0	0	0	0	0	0	0	0
TAGT	1357	2030.4	1963.4	2092.6	0	0.5	0	2	0	0	0	0	0	0	0	0
TATC	6983	7596.3	7481.9	7733.2	12	10.6	5.5	17	0	0	0	0.5	0	0	0	0
TATG	4650	5546.8	5419.4	5655	5	6.6	1.5	12	0	0	0	0	0	0	0	0
TATT	3156	2937.1	2854.8	3023.6	4	2.2	0	5	0	0	0	0	0	0	0	0
TCAA	12234	8861.3	8736	8983	24	16.1	9	24.5	0	0	0	0	0	0	0	0
TCAC	14988	12641.3	12507.9	12769.2	33	13.5	7	19.5	0	0	0	0	0	0	0	0
TCAG	9471	8305.9	8138.6	8450.7	30	20	12	28.5	0	0	0	0.5	0	0	0	0
TCAT	9199	9006	8889.4	9148	15	9.8	5	17.5	0	0	0	0	0	0	0	0
TCCA	8055	9297.7	9159.9	9439.3	15	27.9	19	36	1	0	0	1	0	0	0	0
TCCC	10530	12970	12843.6	13098.5	9	28.3	19	37.1	0	0.1	0	1	0	0	0	0
TCCG	18709	18154.2	17952.9	18353.2	51	77.4	63	92.5	0	0.4	0	2	0	0	0	0
TCCT	7851	11375.5	11251.5	11519	17	24.5	15.5	33	0	0.1	0	1	0	0	0	0
TCGA	26894	20842.5	20649.9	21035.9	142	132.3	111.5	149.1	1	0.2	0	1	0	0	0	0
TCGC	28633	28031.4	27814.6	28228.5	97	105.2	91	123.1	0	0.5	0	2	0	0	0	0
TCGG	30877	27535.2	27251.8	27764.9	168	139.6	122	159.5	1	0.6	0	2	0	0	0	0
TCGT	14255	15734.9	15557.6	15905.6	77	67.4	55	82.5	0	0.2	0	1	0	0	0	0
TCTA	4404	3974.6	3869.9	4067.6	3	6.5	2	11	0	0	0	0	0	0	0	0
TCTG	10528	11283.4	11133	11477.6	19	22.7	15.5	30.5	0	0	0	1	0	0	0	0
TCTT	5704	7289.1	7149.7	7411.3	12	6.2	2	11	0	0	0	0	0	0	0	0

TGAA	6811	9294.3	9159.4	9413.1	2	13.9	7	21	0	0	0	0	0	0	0	0
TGAC	16645	16892.3	16710.4	17094.6	21	29.7	19.5	39	0	0	0	0	0	0	0	0
TGAG	7672	8478.9	8317.3	8642.2	17	21.1	13	28.5	0	0	0	0	0	0	0	0
TGAT	13366	12270.5	12139.5	12401.7	10	27.2	18.5	36	0	0	0	0	0	0	0	0
TGCA	12370	11015.6	10897.9	11134.1	21	24.3	16.5	32.5	0	0	0	1	0	0	0	0
TGCC	22007	18772.9	18570.4	18968	80	56.5	43.5	70.1	1	0.4	0	2	0	0	0	0
TGCG	24107	21081.7	20891.1	21292.7	142	96.1	81.5	113.1	0	0.4	0	2	0	0	0	0
TGCT	19510	15462.4	15271.3	15645.6	27	21	14	30.6	0	0	0	0	0	0	0	0
TGGA	15979	21031.5	20880.7	21231.8	36	53.1	44	64	0	0	0	0	0	0	0	0
TGGC	29567	32095.8	31878.3	32362.1	252	271.1	248.4	299.1	3	2.4	0	6	0	0.1	0	1
TGGG	18503	21527.4	21365.9	21687.9	117	121.5	102	140.6	0	0.5	0	2	0	0	0	0
TGGT	21037	19690.4	19547.7	19838.6	69	67.1	51	82	0	0.3	0	2	0	0	0	0
TGTA	3100	4096.4	4011.7	4185.8	3	3.5	0.5	8	0	0	0	0	0	0	0	0
TGTC	13262	11910.9	11727	12054.6	21	16.8	10.5	24	0	0	0	0.5	0	0	0	0
TGTT	11017	9827	9680.7	9957.3	9	10.2	4	16.5	0	0	0	0	0	0	0	0
TTAA	956	1953.6	1885.4	2025.8	0	0.6	0	2	0	0	0	0	0	0	0	0
TTAC	4057	4791.8	4681	4931.9	4	3.7	1	8	0	0	0	0	0	0	0	0
TTAG	1307	1772.7	1700	1853.5	1	0.6	0	2	0	0	0	0	0	0	0	0
TTAT	2027	2980.4	2887.5	3092.8	2	1.8	0	5	0	0	0	0	0	0	0	0
TTCA	8920	7827.4	7721.4	7952.1	12	9.8	5	15	0	0	0	0	0	0	0	0
TTCC	10617	9132.6	9012.9	9245.8	11	14.8	9	22.5	0	0	0	0.5	0	0	0	0
TTCG	19488	17860.3	17687.4	18028.1	56	42	30.5	52.5	0	0.1	0	1	0	0	0	0
TTCT	6483	6614.6	6505.9	6703.1	9	10	4.5	15.5	0	0	0	0	0	0	0	0
TTGA	7415	8136.9	7979.9	8310.4	10	22.9	14	31.5	0	0	0	0	0	0	0	0
TTGC	12775	11046.2	10857.1	11228.3	32	23.5	16	34.1	0	0	0	1	0	0	0	0
TTGG	12682	14615.9	14441.8	14774.5	38	46.9	35.5	60.6	0	0.2	0	2	0	0	0	0
TTGT	6361	6126.5	6000.8	6262.5	15	14.8	9	21	0	0	0	0	0	0	0	0
TTTA	1632	2314.1	2244.3	2397.2	1	1.7	0	4	0	0	0	0	0	0	0	0
TTTC	8285	6415.6	6266.1	6552	13	7.8	3	14	0	0	0	0	0	0	0	0
TTTG	6120	6789.6	6662.5	6963.7	7	11.5	6	18	0	0	0	1	0	0	0	0

Abbreviations and Notes: Nuc: Nucleotide; Len: Length; Obs: Observed; Exp: Expected; PCTL: Percentile. Tetranucleotide repeats that are at the same time mono- or dinucleotide repeats (for example, AAAA and ACAC), are not listed.

1E) Observed and Expected Number of Mononucleotide Repeats (with 2.5 and 97.5 Percentile from 100 Randomizations) in *M. leprae*

	A				C				G				T			
Len	Observed	Expected	2.5 PCTL	97.5 PCTL	Observed	Expected	2.5 PCTL	97.5 PCTL	Observed	Expected	2.5 PCTL	97.5 PCTL	Observed	Expected	2.5 PCTL	97.5 PCTL
1	198118	195447.9	195049.15	195983.33	260086	247530.68	246946.13	248243.45	256783	247955.75	247351.1	248579.9	206686	207338.11	206734.38	207965.58
2	42812	42641.39	42385.85	42903.78	75878	74081.86	73732.08	74496.28	88749	87968.02	87640.88	88317.83	45330	44821.29	44492.83	45088.15
3	7160	7895.39	7785.43	8036.88	14630	17107.44	16863.85	17279.75	17521	19044.13	18840.23	19280.1	8130	8389.85	8238.43	8555.58
4	1596	1750.84	1696.33	1811.63	2591	3852.04	3742	3942.58	3621	4099.53	3982.43	4208.53	1750	1776.24	1693.38	1866.53
5	404	404.94	371.43	436.05	440	893.91	843.85	949.1	671	1146.77	1093.03	1209.63	518	376.12	341	408
6	74	89.95	74.48	104.53	51	206.09	183.95	231.53	87	268.07	237.9	300.63	74	87.7	73.48	104.05
7	13	21.1	13	29	13	51.25	38.48	63.53	17	63.52	48	79.05	10	19.33	12	28.53
8	4	5.46	1	9.52	0	13.62	7.48	21	6	19.58	12	26.53	2	3.84	1	7
9	0	0.86	0	3	0	4.26	0	7.52	0	5.48	2	10.53	0	1.09	0	3
10	0	0.19	0	1	0	1.15	0	3	0	1.62	0	4	0	0.11	0	1
11	0	0.02	0	0	0	0.44	0	2	0	0.52	0	2	0	0.06	0	1
12	0	0.03	0	0.52	0	0.26	0	2	1	0.25	0	1	0	0	0	0
13	0	0	0	0	0	0.06	0	1	0	0.1	0	1	0	0	0	0
14	0	0	0	0	0	0.04	0	1	0	0	0	0	0	0	0	0
15	0	0	0	0	0	0.01	0	0	0	0	0	0	0	0	0	0
16	0	0	0	0	0	0.02	0	0	0	0	0	0	0	0	0	0
17	0	0	0	0	0	0.03	0	0.52	0	0	0	0	0	0	0	0
18	0	0	0	0	0	0	0	0	0	0	0	0	0	0	0	0

1F) Observed and Expected Number of Mononucleotide Repeats (with 2.5 and 97.5 Percentile from 100 Randomizations) in *E. coli*

	A				C				G				T			
Len	Observed	Expected	2.5 PCTL	97.5 PCTL	Observed	Expected	2.5 PCTL	97.5 PCTL	Observed	Expected	2.5 PCTL	97.5 PCTL	Observed	Expected	2.5 PCTL	97.5 PCTL
1	479679	486296.42	485509.33	487087.65	607157	583448.38	582591.15	584556	619905	612541.84	611800.75	613671	531299	535369.29	534315.95	536319
2	133515	133725.83	133307.25	134240	150828	149006.77	148602.48	149863	185052	180014.87	179473.3	180698	133886	136789.4	136359.98	137670
3	45113	45147.73	44892.78	45499	25839	31359.46	31091.1	31633	30719	34804.83	34581.18	35071	40291	36395.17	36162.9	36750
4	14646	13322.05	13146.43	13520	3224	5347.85	5219.7	5579	7022	6974.37	6834.75	7166	10546	10569.27	10411.23	10868
5	6095	5225.83	5143.38	5383	496	806.82	755.48	884	1198	1844.18	1779.43	1954	3149	3030.59	2929.45	3182
6	1967	1978.83	1917.43	2061	68	158.76	136	187	151	391.23	357	431	808	936.68	888.48	1014
7	375	518.8	482.48	566	9	29.04	20	40	28	89.11	69	110	170	287.55	253.48	312
8	76	162.78	142.95	188	1	6.05	2	14	4	25.34	17.48	40	22	81.96	66.95	99
9	3	56.1	45	70	0	1.41	0	5	0	5.85	2	13	2	22.86	15	31
10	0	15.54	9.48	27	0	0.42	0	2	0	1.41	0	6	0	5.37	1	12
11	0	3.76	1	9	0	0.1	0	1	0	0.58	0	3	0	1.94	0	5
12	0	1.31	0	4	0	0	0	0	0	0.19	0	2	0	0.47	0	3
13	0	1.14	0	3	0	0	0	0	0	0.02	0	1	0	0.27	0	2
14	0	0.24	0	2	0	0	0	0	0	0	0	0	0	0.01	0	1
15	0	0	0	0	0	0	0	0	0	0	0	0	0	0.09	0	1
16	0	0	0	0	0	0	0	0	0	0.02	0	1	0	0	0	0
17	0	0	0	0	0	0	0	0	0	0	0	0	0	0	0	0
18	0	0	0	0	0	0	0	0	0	0	0	0	0	0	0	0
