# Supplementary material for: Stabilization of the genome of the mismatch repair deficient Mycobacterium tuberculosis by context-dependent codon choice
Source: BMC Genomics. 2008 May 28;9:249. doi: 10.1186/1471-2164-9-249 (PMC2430213; doi:10.1186/1471-2164-9-249)
Supplement: Additional file 2 — Tables with estimated frame-shift mutation rates in mononucleotide repeats in protein coding genes of M. tuberculosis, M. leprae and E. coli. Estimates are given for the real genomes as well as for randomized genomes that preserve the amino acid sequence and the gene-specific codon frequencies. [file 1471-2164-9-249-S2.pdf]

Additional File 2

Estimated Frame-shift Mutation Rates in Mononucleotide Repeats

2A) *M. tuberculosis*

	c = 2			*c* = 2.5			*c* = 3			*c* = 3.5			*c* = 4		
i	Obs	Exp	Exp/Obs	Obs	Exp	Exp/Obs	Obs	Exp	Exp/Obs	Obs	Exp	Exp/Obs	Obs	Exp	Exp/Obs
4	0.0025	0.0048	1.9141	0.0018	0.0069	3.7913	0.0014	0.0301	20.8451	0.0012	0.1942	159.2271	0.0011	1.1240	1042.6580
5	0.0009	0.0028	3.1354	0.0008	0.0056	7.1071	0.0007	0.0293	39.9826	0.0007	0.1936	278.3949	0.0007	1.1235	1660.8235
6	0.0003	0.0016	6.2077	0.0003	0.0047	16.6473	0.0003	0.0284	93.6947	0.0003	0.1929	587.3751	0.0004	1.1229	3159.6869

2B) *M. leprae*

	c = 2			*c* = 2.5			*c* = 3			*c* = 3.5			*c* = 4		
i	Obs	Exp	Exp/Obs	Obs	Exp	Exp/Obs	Obs	Exp	Exp/Obs	Obs	Exp	Exp/Obs	Obs	Exp	Exp/Obs
4	0.0008	0.0013	1.6558	0.0006	0.0015	2.2627	0.0007	0.0034	5.0003	0.0009	0.0127	13.4171	0.0016	0.0485	30.6961
5	0.0003	0.0007	2.3559	0.0003	0.0011	3.3047	0.0004	0.0031	6.9117	0.0008	0.0125	16.0591	0.0015	0.0484	33.3114
6	0.0001	0.0004	3.6528	0.0002	0.0008	4.8393	0.0003	0.0029	9.0260	0.0007	0.0123	18.4589	0.0014	0.0482	35.5780

2C) *E. coli*

	c = 2			*c* = 2.5			*c* = 3			*c* = 3.5			*c* = 4		
i	Obs	Exp	Exp/Obs	Obs	Exp	Exp/Obs	Obs	Exp	Exp/Obs	Obs	Exp	Exp/Obs	Obs	Exp	Exp/Obs
4	0.0020	0.0027	1.3161	0.0016	0.0025	1.6113	0.0013	0.0035	2.6622	0.0012	0.0079	6.8039	0.0011	0.0245	22.3482
5	0.0009	0.0014	1.5720	0.0008	0.0017	2.0530	0.0008	0.0029	3.6544	0.0008	0.0075	9.4892	0.0008	0.0242	29.8556
6	0.0004	0.0007	2.1132	0.0004	0.0012	2.9620	0.0004	0.0025	5.6280	0.0005	0.0072	14.6307	0.0005	0.0239	43.8279

Abbreviations and Notes: 'Obs' refers to the estimated genomic frame-shift mutation rates in mononucleotide repeats in protein-coding genes in the real genomes of *M. tuberculosis, M. leprae* and *E. coli.* 'Exp' refers to the mean estimated genomic frame-shift mutation rates in mononucleotide repeats in protein-coding genes in randomized genomes of these organisms (based on 100 randomizations; the randomized genomes had the same amino acid sequence and the same gene-specific codon usage as the real genomes, as explained in the methods).

The parameter c refers to the factor by which the frame-shift mutation rate in a mononucleotide repeat increases with each nucleotide added to the repeat. The parameter i refers to the minimal length needed for a mononucleotide repeat to exhibit a significant frame-shift mutation rate. For details see Methods.
